# Uncontrolled pain: a call for better study design

**DOI:** 10.3389/fvets.2024.1328098

**Published:** 2024-02-14

**Authors:** Timothy H. Hyndman, Ross S. Bowden, Andrew P. Woodward, Daniel S. J. Pang, Jordan O. Hampton

**Affiliations:** ^1^School of Veterinary Medicine, Murdoch University, Murdoch, WA, Australia; ^2^Harry Butler Research Institute, Murdoch University, Murdoch, WA, Australia; ^3^School of Mathematics, Statistics, Chemistry and Physics, Murdoch University, Murdoch, WA, Australia; ^4^Faculty of Health, University of Canberra, Canberra, ACT, Australia; ^5^Faculty of Veterinary Medicine, University of Calgary, Calgary, AB, Canada; ^6^Faculty of Science, University of Melbourne, Parkville, VIC, Australia

**Keywords:** animal ethics, study design, randomized controlled trials, analgesia, farm animals

## Abstract

Studies assessing animal pain in veterinary research are often performed primarily for the benefit of animals. Frequently, the goal of these studies is to determine whether the analgesic effect of a novel treatment is clinically meaningful, and therefore has the capacity to improve the welfare of treated animals. To determine the treatment effect of a potential analgesic, control groups are necessary to allow comparison. There are negative control groups (where pain is unattenuated) and positive control groups (where pain is attenuated). Arising out of animal welfare concerns, there is growing reluctance to use negative control groups in pain studies. But for studies where pain is experimentally induced, the absence of a negative control group removes the opportunity to demonstrate that the study methods could differentiate a positive control intervention from doing nothing at all. For studies that are controlled by a single comparison group, the capacity to distinguish treatment effects from experimental noise is more difficult; especially considering that pain studies often involve small sample sizes, small and variable treatment effects, systematic error and use pain assessment measures that are unreliable. Due to these limitations, and with a focus on farm animals, we argue that many pain studies would be enhanced by the simultaneous inclusion of positive and negative control groups. This would help provide study-specific definitions of pain and pain attenuation, thereby permitting more reliable estimates of treatment effects. Adoption of our suggested refinements could improve animal welfare outcomes for millions of animals globally.

## 1 Introduction

Imagine a study where the goal is to assess the effectiveness of a new analgesic drug for use in cattle. Assume the analgesic has relatively few adverse side effects and is cost-effective in clinical applications. If shown to be sufficiently effective, this analgesic will likely be adopted and used on millions of cattle for routine husbandry procedures that are painful, such as surgical castration. You design a trial to assess the analgesic efficacy of this new drug in young bulls. In your trial, you will have one control group of bull calves that will be castrated and receive the gold standard analgesic (the most effective analgesic). You have a second group, the experimental group, that will also be castrated but will receive the new analgesic. The statistical analysis of the trial shows that the pain in both groups was similar. With this evidence that the new analgesic performed similarly to the gold standard, it is subsequently adopted and utilized for painful procedures in millions of young bulls.

Consider an alternate design with a third group of cattle where no analgesia is provided at the time of castration. You compare the pain in all three groups and find it is similar. Interpretation of the results is now more complicated than before because the outcome for the gold standard analgesic is now similar to no-analgesia. Perhaps the method of pain assessment was inadequate, meaning potentially relevant differences between the three groups was lost in the noise of the pain assessment results. That the pain response was similar between the gold standard analgesic and the novel analgesic is *consistent* with effectiveness of the novel analgesic, but not necessarily *evidence of* its effectiveness. Though the novel analgesic may have been similar to the gold standard in this study, without the no-analgesic control group, it is unwarranted to conclude that it is superior to doing nothing. If it in fact *is not* efficacious, and it is adopted for widespread use, then potentially millions of cattle will continue to suffer from the pain associated with castration. Worse, it may be presumed that these cattle are receiving effective pain relief from the new analgesic, which may dissuade others from further investigation into this area.

The central point here is that without a no-analgesia control group, erroneous conclusions with quantitatively enormous implications can easily be reached. Here, we dissect the need for pain in order to scientifically assess response to potential analgesics in animals that are used in veterinary research; research primarily for the benefit of animals. The purpose of this discussion is not to focus on the validity of statistical methods chosen to analyse data arising from pain experiments. Rather, the focus is on study design. To address this issue, we first review the background to modern pain research in animals.

## 2 Animal use committees

In most post-industrial nations, the use of animals for research, unlike most other forms of animal use, requires legal permission. To attain this permission, researchers must first submit an application with a detailed description of the project to an institutional animal use committee (1). These have various names globally, such as Institutional Animal Care and Use Committees (United States), Animal Care Committees (Canada), and Animal Ethics Committees (Australia and New Zealand) (2). Animal use committees assess applications proposing the use of animals for research through consideration of what might justify deliberately causing harm to sentient animals. This necessitates the application of ethical theories to these often-contentious questions (3). The ethical principles underpinning scientific animal use have been evolving over the last century, noting there are significant regional differences when it comes to the extent of the requirements made on researchers (4). The source of animals can also influence the decision-making process of an institutional animal use committee. Important distinctions are sometimes made between the use of pet, laboratory, farm or wild animals (5, 6). Our discussion will primarily consider farm animals but many of the principles discussed are relevant to all animal pain research.

## 3 Research ethics

When considering the use of animals for research purposes, the first requirement is that scientists should strive to minimize harm, typically expressed as the so-called “3Rs” (7), i.e., ways to Reduce the number of animals used to the minimum necessary for scientific validity; to Replace experiments that use live animals with alternative methods; and to Refine procedures for the remaining experiments so as to minimize suffering (8).

The second requirement is that animals should be used only when that use is likely to give rise to genuine benefits to humans (or animals) (9). Put another way, committees are expected to ensure that there is a proper balance between the harm imposed upon animals and the expected benefits of causing that harm. This is known as harm-benefit analysis, and is the core consequentialist ethical underpinning of animal-based research (10). The 3Rs have been implemented as an integral part of the way animal experiments are regulated and reviewed in many countries, and a requirement for consideration of harm-benefit analysis is also widely, but not universally, practiced (11).

The third requirement is to put an absolute cap on the suffering that animals may endure in the course of an experiment. According to this requirement, experiments should not be allowed if they involve *severe* suffering. Of course, this requirement could be seen as a special case of the requirement to Refine procedures. This is because the requirement to Refine is relative to what is possible without sacrificing the goal of the research, but the requirement to avoid severe suffering is absolute. This requirement has, with some limitations, been adopted by the European Union but has so far had little uptake outside Europe (12). All such restrictions placed on animal use must be applied in a way that still allows scientifically robust results to be produced from research, and that requires statistical validity.

## 4 Sample size

There are a litany of critically-important aspects to sound study design such as randomization, blinding and data handling (13–16) but animal use committees will also consider the necessity of an appropriate sample size before animal-based studies can commence. Studies must be sufficiently large that their estimates are not overly influenced by random variation (17) and to reduce the occurrence of overestimates caused by chance (18). At the same time, researchers are expected to use the fewest animals capable of answering their question under the 3Rs principle of Reduction (19). This means that there is an unavoidable trade-off to achieve a sufficient but not excessive sample size (20). If Reduction is attempted without consideration for statistical rigour, a relatively small number of animals may be harmed, but it is likely that nothing useful will be learned. These ethical concerns become especially heightened when *painful* things are done to animals.

## 5 Pain and clinical research

Pain is defined in several ways that are generally variations on the theme of “an unpleasant sensory and emotional experience associated with, or resembling that associated with, actual or potential tissue damage” (21). There is a great deal of variation in how animals display signs of pain (22) and given one aspect of it is emotion, assessing it objectively is challenging, especially in species that have evolved to hide or ‘mask’ signs of overt pain, such as ruminants (23). Before we explore the details of study design in pain research, a few definitions are necessary.

There are important differences between *hypo*algesia and *an*algesia with the former being an attenuation of pain and/or nociception whereas the latter is complete ablation of pain (24). For the purposes of this discussion, the terms analgesia and hypoalgesia are considered synonymous. For pain-based research, there is nearly always the requirement for an ‘intervention’, defined here as treatments applied to *attenuate* pain (e.g., administering an analgesic). We are not using the term ‘intervention’ to describe the procedure that *causes* pain in studies where the pain is experimentally-induced.

Then, there is the concept of a result being *clinically-relevant*. Statistical significance refers to whether a value of p is below a chosen level (typically *p* < 0.05) and is often used to determine differences between study groups, but it does not quantitate those differences. Knowing the magnitude of those differences is crucial when deciding whether that difference is clinically-relevant (25). While statistical methods can quantify what occurred, perceptions of clinical relevance are informed by values and norms, and require subjective value judgments. For example, small differences seen between an experimental group and a control group may be statistically significant but the difference may be clinically meaningless. Clinical relevance, and not statistical significance, generally informs clinical practice standards and policy decisions (26). Moreover, increasing calls are being made to stop using statistical significance and hypothesis testing as the primary tool from which a study draws its conclusions. This is due to the arbitrary nature of the convention to have *p* < 0.05 (or any other value) define the level of significance, and because many studies (especially in the biomedical sciences) have small and variable effects, systematic error and noisy measurements (27, 28). The reporting of estimated effect sizes is included in the guidelines of the Consolidated Standards of Reporting Trials (CONSORT) (29).

Policy decisions, such as standards imposed on agricultural producers for routine husbandry practices in farm animals, have the capacity to impact upon orders of magnitude more animals than were involved in any study assessing these practices. For example, sample sizes of ~10 are not uncommon for studies in farmed chickens that assess pain associated with slaughter procedures (30). The results of these studies may then be applied to the billions of chickens that are slaughtered for human consumption every year (31). Therefore, scientific rigour in animal pain studies (especially in livestock) is extremely important for shaping practices that affect large numbers of animals.

## 6 Pain study design

**Figure 1:**
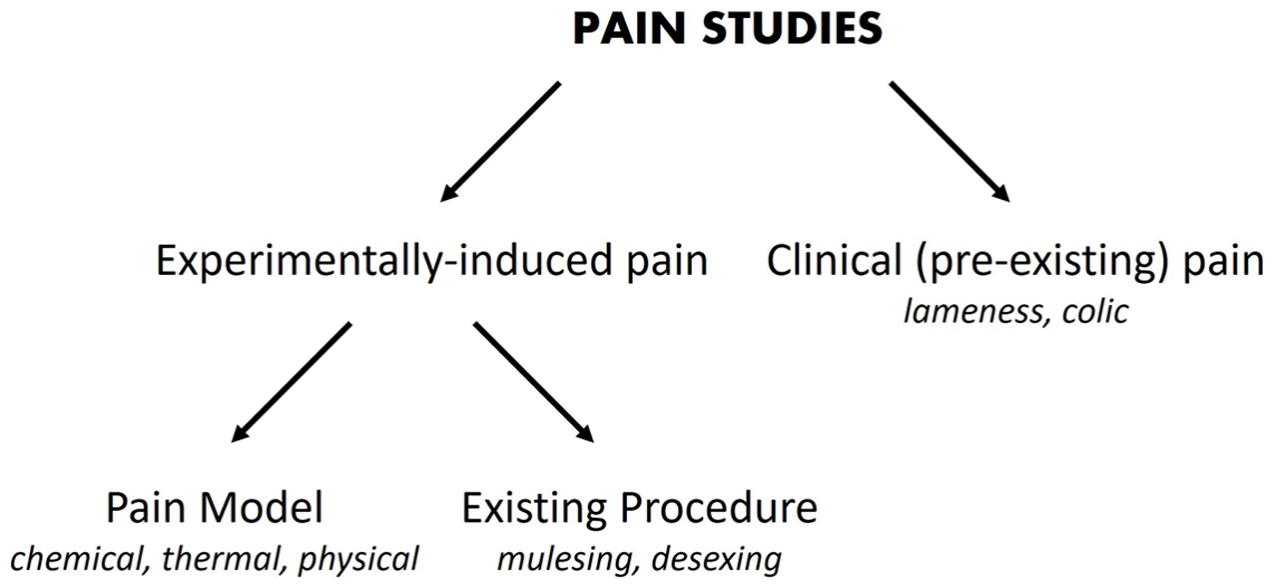
Pain studies may experimentally-induce pain or utilize pain that is already present. Examples or categories of each pain source are provided in italic font.

Investigators may be motivated to perform a pain study for a variety of reasons. There may be an imperative to alleviate pain following a common surgical procedure [e.g., desexing (32)] or a routine husbandry practice [e.g., mulesing (33)], or the primary interest may be in an analgesic that could be used for many causes of pain (34). Regardless of the impetus for the study, a number of choices need to be made about how the study will be designed. The source of pain (Figure 1) and a suitable way to measure it need to be chosen.

The source of pain may be pre-existing and will be seen commonly in clinical practice (35). Examples include pain arising from lameness (36) and colic (37). Pain may also be experimentally-induced, which can be subdivided into pain caused using standardized approaches (pain models), where the noxious stimulus may be chemical (e.g., formalin, capsaicin), thermal (e.g., heat probes) or physical (e.g., skin incisions) (34, 38), or pain that is induced by existing procedures such as mulesing or surgical desexing.

The method of pain assessment plays an important part in interpreting the results of a pain study. Surrogate measures are often used, especially in animals, as pain is difficult to quantify. Examples include force plate analysis for lameness studies (39), nociceptive threshold testing using a noxious thermal stimulus (40) and the evaluation of acute pain by electroencephalograms following noxious chemical stimulus (41). The quantification of pain will rely on methods that were validated previously, or are validated as part of the study (42). Ideally, pain assessment should be at least species-specific, context-specific, validated and potentially composite (using a suite of measures) (22, 43–45). For simplicity, this discussion will assume that a single outcome measure of pain has been used in a study and that different levels of pain can be distinguished.

## 7 Control groups

To determine the effect that an intervention (e.g., a novel drug) has on pain, comparisons should be made between a group of study participants that received this intervention (the experimental group) and one or more other groups that provide measures of analgesia and/or no analgesia [the control group(s)] (46). Where the intervention is a drug, there should be evidence that it has an acceptable safety profile in the target species. For a study that contains its own control group(s), the study is internally controlled and inferences can be made about its internal validity (47), such as were the methods used in that study capable of detecting pain at all? Where a study is uncontrolled, control groups from other studies are often used, which are referred to as external, or historical, controls; noting that the use of historical controls is often prone to bias (48, 49). For studies that have a single control group, the control group may be a *positive control* or a *negative control* (50). For a pain study, a positive control involves the provision of a drug or method that provides measurable and meaningful pain attenuation. A negative control represents the absence of any pain attenuation methods. In drug trials, placebos are often used for the negative control (51), noting that a placebo control group should not be considered interchangeable with a no treatment group (52). The same can be said about sham-procedures being different to no intervention (53, 54).

Before beginning a study that compares pain in an experimental group to pain in a single control (positive or negative) group, it will not be known whether there will be a clinically-relevant difference between the two groups (if it *was* known, there would not be scientific motivation to do the study). This means the investigators need to consider the possibility that there will not be a clinically-relevant difference.

For single control group studies, there are three possible outcomes – the outcome measure of pain in the experimental group can be below, above, or not relevantly different to the outcome measure from the control group. For the scenarios where the outcome measure is above or below the outcome measure of the control group, it will often be assumed that because a (statistically) clear result was obtained, the methods of pain assessment were appropriate to distinguish the groups in a way that then permits a judgment as to whether that difference is clinically-relevant. When there is a null result (i.e., no meaningfully-significant difference between the two groups), the interpretation of the results needs to be more nuanced (55). A true difference between the groups may have been missed due to an inadequate sample size that meant imprecise estimates of the outcome measure in one or both groups prevented the true difference from being distinguished from experimental noise (56).

**Figure 2:**
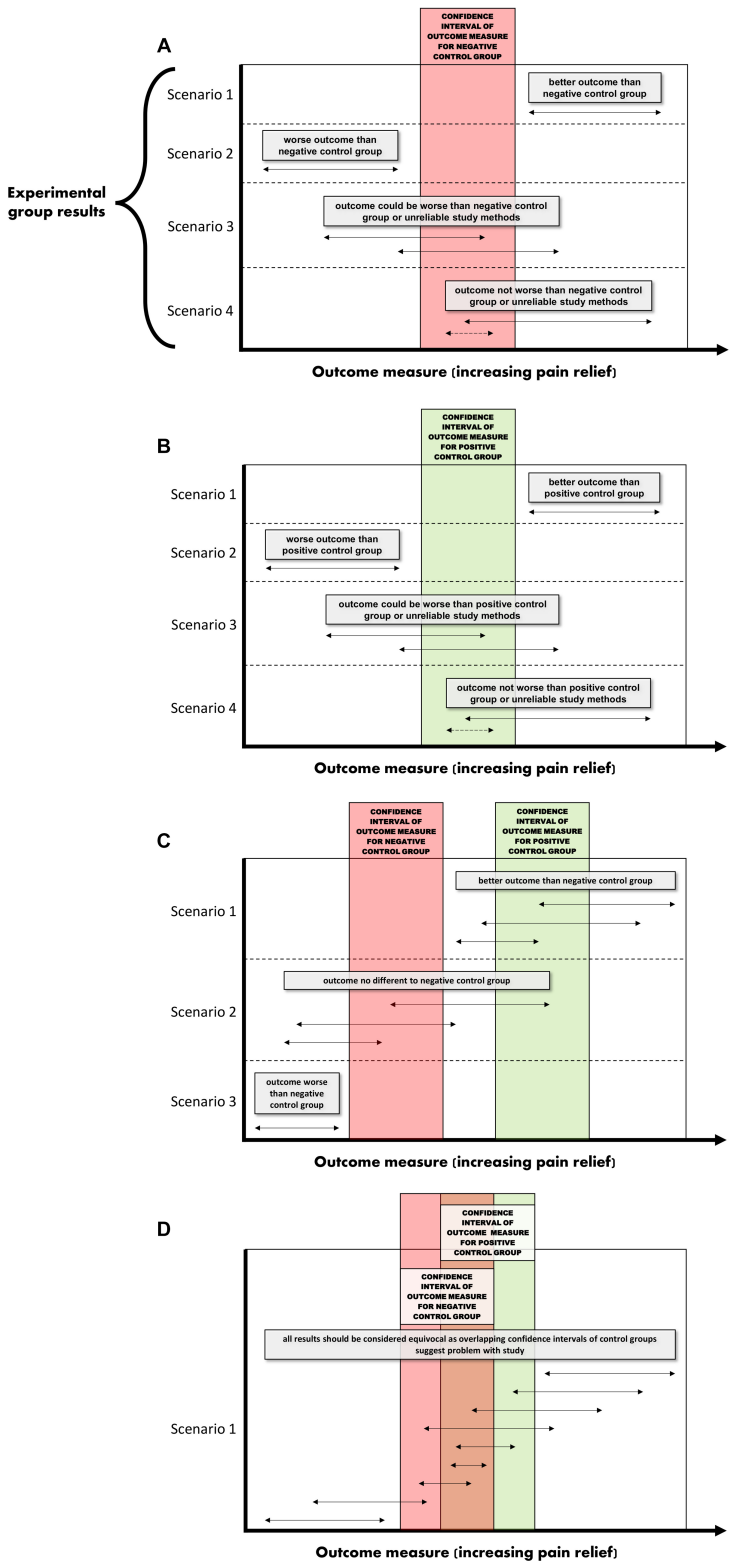
**(A–D)** Interpretations for hypothetical scenarios where the outcome measure of pain in the experimental group (represented as arrows to reflect confidence intervals) exceeds, is no different, or is below the confidence interval of the outcome measure of pain in the control group(s). Interpretations are included in grey text boxes that overlay each plot and are made in the context of whether the study has a negative control group only (plot **A**), a positive control group only **(B)**, negative and positive control groups that have non-overlapping results **(C)**, or negative and positive control groups with results that overlap **(D)**. The confidence intervals are assumed to be constructed such if they do not overlap then this indicates a statistically significant difference.

In a negative (placebo)-controlled study (Figure 2A), a null result is more likely to occur if the assessment of pain is unreliable (and is without bias toward either group). Assuming the experimental intervention truly provides some level of pain relief, and if unreliable methods are the cause of the null result, then the conclusion that the intervention used in the experimental group does *not* provide analgesia, will be false.

In studies controlled only by a positive control group, it is tempting to conclude from a pain study that compares a novel intervention in the experimental group to only a positive control group that a null result (Scenarios 3 and 4 in Figure 2B) reflects equivalence to a positive control; ergo, the novel intervention in the experimental group decreased pain. However, this conclusion is based on an assumption that the positive control is itself superior to a negative control, the so-called ‘historical control assumption’ (57). The method of pain assessment also needs to be considered. If it is unreliable (and unbiased), then as for the negative-controlled trial, a null result of no relevant difference becomes more likely. This immediately clouds the capacity to distinguish between (a) the true absence of any relevant difference between the experimental group and the positive control group, and (b) whether the method of pain assessment was unreliable. If it is assumed that the intervention does not provide pain relief (and therefore there is truly a difference between the experimental group and the positive control group), then if unreliable methods of pain assessment were the cause of a null result (i.e., the methods have prevented a relevant difference being observable), then the conclusion that the novel intervention *did* work will be false.

## 8 Discussion

### 8.1 Suggestions for improvement

So what does this all mean? If a study only includes a single control group, and if a statistically significant difference is observed between the experimental group and the control group, it provides some evidence that the pain assessment methods used were sufficient to reach the conclusion that there is a difference between the two groups (Scenarios 1 and 2 for Figures 2A,B). In strong contrast to this, when there is no difference between the experimental group and the single control group, then there are two conclusions that cannot be easily distinguished; either the intervention in the experimental group performs similarly to the control, or that the methods of pain assessment were inadequate (Scenarios 3 and 4 for Figures 2A,B).

Confidence in the study conclusions will be enhanced if the study utilises pain assessment methods that have been validated in previous studies (e.g., those reviewed by Muley, Krustev and McDougall (58) for experimental models of inflammatory pain). Adoption of these validated methods in a study with only one control group will offset some (but not all) of the concerns of the diminished internal validity of the study (i.e., by not having positive *and* negative control groups). However, caution needs to be exercised as some of these methods have been shown to have limited reproducibility, or are not broadly translatable (59).

What is more resilient to scientific criticism than using a single control group with a validated method, is the inclusion of a positive control group *and* a negative control group (Figure 2C) (46, 60–65). The inclusion of both a positive and a negative control group provides an opportunity to assess the internal validity of the method of pain assessment (Figures 2C, D). That is, is the study of sufficient internal validity that it can provide study-specific definitions of pain attenuation and no pain attenuation? If so, conclusions can be more confidently drawn about the pain attenuating effect of the novel intervention by comparing the experimental group to these two control groups.

There are certain situations where single-controlled studies may still provide study-specific definitions of pain attenuation and no pain attenuation, while also providing an assessment of the internal validity of a study (Table 1). As described earlier, the source of pain in a study may be experimentally-induced or pre-existing (Figure 1). If pain is experimentally-induced in pain-free animals, then including a negative (placebo) control group would provide a study-specific definition of pain following pain induction (66, 67). Those animals that received the novel intervention could be compared to this negative control group to see if there was pain attenuation, but comparisons between the experimental group and a ‘gold standard’ level of pain attenuation are absent in this study as there is no positive control group. In contrast, if the pain is pre-existing, then including a positive control group would provide a study-specific definition of pain attenuation (68). Those animals that received the novel intervention could be compared to both the positive control group and their own pre-intervention pain outcome measures to see if there was pain attenuation. However, the absence of a negative control group means that clinical pain that naturally subsides (after the novel intervention is administered) is harder to identify, and the natural phenomenon of ‘regression to the mean’ remains a problem (69). As a generalization for single-controlled studies, negative-controlled studies are better suited for experimentally-induced pain, and positive-controlled studies are better suited for pre-existing pain.

**Table 1:** Control groups used in pain studies according to the source of pain.

Study type	Positive-control group^a^	Negative (placebo)-control group^b^	Source of pain			
			Clinical pain (pre-existing pain)		Experimentally-induced pain	
Double-controlled study	Yes	Yes	The novel intervention is compared to study-specific definitions of no analgesia and effective analgesia. As the pain was pre-existing, there is no definition of pain-free.	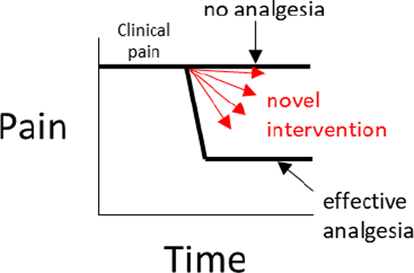	The novel intervention is compared to study-specific definitions of no analgesia and effective analgesia. As the pain was induced, baseline measurements can define pain-free.	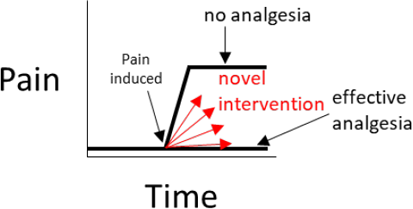
Positive-controlled study	Yes	No	The novel intervention is compared only to effective analgesia. There is no negative control group to control for clinical pain that naturally subsides.	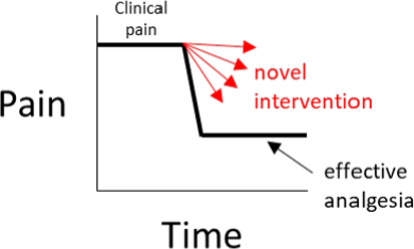	The novel intervention is compared only to effective analgesia. There is no negative control group to permit measurement of no pain attenuation or to assess whether pain was induced at all (this assumes the positive control was highly effective).	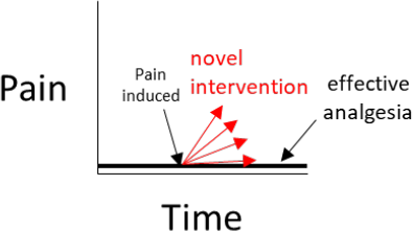
Negative- controlled study	No	Yes	The novel intervention is compared only to no analgesia. There is no positive control group to permit measurement of pain attenuation.	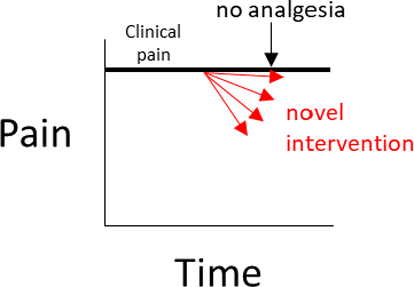	The novel intervention is compared only to no analgesia. There is no positive control group to permit measurement of pain attenuation.	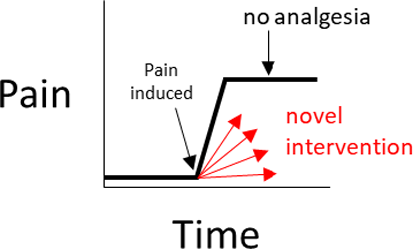

Novel interventions are compared to negative and/or positive controls. Novel interventions may include surgical procedures and drugs where the pain-attenuating effect of the intervention is unknown. ^a^Positive controls may include established surgical procedures and analgesic drugs that are known to provide pain relief. ^b^Negative controls are known to not provide any pain relief and may include the deliberate omission of any intervention or the administration of a placebo.

To this point, it has been assumed that a positive control is available for a pain study. But what if it is not? Remember that the purpose of the positive control is to provide a study-specific definition of pain attenuation. The corollary to this is that the positive control provides a level of analgesia that is meaningfully different to doing nothing. Therefore, positive controls can be found by comparing potential analgesics to negative controls. A value judgment can then be used as to whether the treatment effect of the positive control will make it appropriate to use as a positive control in studies assessing other potential analgesics. In other words, the newly discovered positive control may outperform doing nothing, but is its analgesic efficacy clinically relevant? If it is, then its ongoing use as a positive control is more easily justified for studies that intend to assess pain in the same context (e.g., where the target species, source of pain and pain measurement method are the same). From a set of positive controls, the so called ‘gold standard’ (or criterion standard) can be defined as the one with an acceptable safety profile and the greatest analgesic efficacy. It should be noted that a ‘gold standard’ would not necessarily be used routinely in field settings as it may be cost prohibitive or not registered in a particular species.

It should be noted that there are other methods that can be used to improve the credence of the study conclusions and/or reduce animal suffering. Bayesian analyses can include so-called prior knowledge (produced by other studies) about the expected outcomes of the positive or negative controls (70). Adaptive study designs are where the design of the study is altered (adapted) after experimentation has started (71). For example, response-adaptive randomization is where the treatment allocation ratio(s) is changed in favor of an intervention with demonstrated beneficial effects (72); or the study is stopped early following an interim analysis that concludes there is sufficient evidence to answer the research question, e.g., a sufficiently precise estimate of the treatment effect has been obtained (73).

### 8.2 Animal welfare harms and benefits

For pain studies, the inclusion of a positive control (analgesia) group is easily justified if an analgesic is available with clinically-relevant effects that can be reliably quantified by the methods being used in the study. The inclusion of a negative (placebo) control (no-analgesia) group is more problematic because this raises the obvious conflict of balancing the ethical use of research animals against the scientific rigour of the studies they are used for. In humans, ethical objections have been raised to this practice, especially in neonates (74, 75). However, the World Medical Association Declaration of Helsinki justifies the use of a placebo control group if there are “compelling and scientifically sound methodological reasons the use of any intervention less effective than the best proven one, the use of placebo, or no intervention is necessary to determine the efficacy or safety of an intervention” (76). For the majority of pain studies in animals, we argue that there *are* “compelling and scientifically sound methodological reasons” to include positive and negative (no analgesia) control groups. This is especially pertinent where study results will be applied to very large numbers of animals, such as farm animals.

Numerous husbandry practices in livestock management are considered to impose unacceptable pain levels by the standards set by animal use committees or scientific journals. Various arguments have been raised to support the position that analgesia is not required or justified for these procedures (77–79). We acknowledge that the validity of these arguments will rest upon chosen ethical theories, and accompanying ethical commitments and priorities. Nonetheless, some of these reasons may be regarded by some as fallacious (or at least ethically contestable), but some are more widely understandable: the financial value of the farm animal relative to the cost of treatment; a belief that the procedure is not painful; the assertion that young animals feel less pain than adults; logistics of drug administration; a limited understanding of farm animal pain behaviors; a fear of adverse side effects following the provision of analgesia; a limited number of analgesics approved for use in farm animals; and worry about violating food withholding periods resulting in drug residues entering the food chain. As a consequence, massive numbers of farm animals continue to be exposed to painful husbandry procedures—such as debudding, dehorning, hoof trimming, castration, tail docking, ear tagging, ear notching and mulesing—without the provision of analgesia (80–84).

Rather than preventing these (likely) painful husbandry practices from being included in pain assessment studies as comparator groups, we argue that there should be encouragement to do so, to facilitate direct comparisons with alternative methods being investigated. These existing husbandry practices are already being done on large numbers of animals and so utilising some of these animals in experimental or observational studies is a pragmatic approach for performing translational research that is likely to be adopted by industry. This amounts to applying a knowledge-based ethic to learn as much as possible from existing practices (85). In more simple terms, it could be called making the best of a bad situation.

Enumerating the animal welfare implications of our proposed approach is worth exploring. Returning to the hypothetical case study with which we began this paper – we are studying a new analgesic for use in farmed cattle. But this time, we assume that the newly developed analgesic (referred to herein as the experimental analgesic) is truly capable of reducing pain by 50% in animals but this effect has not yet been established by controlled studies. A study is proposed that includes a positive control group where animals receive an analgesic known to be effective (herein referred to as the control analgesic) but is prohibitively expensive for large-scale management practices. The use of a negative (no analgesia) control group is not allowed due to the decision of an animal use committee or the editorial policy of a journal. Furthermore, assume that the pain attenuation observed in the experimental group (given the experimental analgesic) was below that for the positive control group (given the control analgesic). Without a negative control group, it is unclear whether the experimental analgesic did indeed attenuate pain, but less so than the control analgesic, or whether there was no observed analgesic effect at all. Without an appreciation of the important distinction that exists between these two interpretations, it is conceivable that the investigators and/or the industry leaders may (erroneously) conclude that the experimental analgesic should not be used and the industry maintains its position to perform the procedure without any analgesia at all.

The animal welfare cost of the experimental analgesic remaining unused increases the pain experienced by millions of cattle; it is now twice as much compared to if the experimental analgesic was used: this is the forgone benefit from the harm-benefit analysis. Thought of another way, this is the animal welfare cost of *not* using the negative control group. The animal welfare benefit of the decision to disallow an observational negative control group is zero – the animals were subjected to the painful procedure anyway as part of routine management. Clearly, the animal welfare outcomes of the decision to disallow a negative control group are profoundly negative.

## 9 Conclusion

For situations where there is a reluctance to include a negative control group, we argue that better net animal welfare outcomes will usually result if well-designed studies harm a (relatively) small number of animals through the inclusion of this group. We acknowledge that researchers need to protect their reputations and preserve ‘social licence’ in an era where animal welfare scrutiny from society can be intense, but that should not come at the cost of scientific rigor. Any attempt to compromise the statistical robustness of pain studies in the name of animal welfare may instead result in worsened animal welfare outcomes for millions of animals.

## Author contributions

TH: Conceptualization, Writing – original draft, Writing – review & editing. RB: Writing – review & editing. AW: Writing – review & editing. DP: Writing – review & editing. JH: Conceptualization, Writing – original draft, Writing – review & editing.
